# Smart Face Mask with an Integrated Heat Flux Sensor for Fast and Remote People’s Healthcare Monitoring

**DOI:** 10.3390/s21227472

**Published:** 2021-11-10

**Authors:** Marc Lazaro, Antonio Lazaro, Ramon Villarino, David Girbau

**Affiliations:** Department of Electronics, Electrics and Automatic Control Engineering, Rovira i Virgili University, 43007 Tarragona, Spain; marc.lazaro@urv.cat (M.L.); ramon.villarino@urv.cat (R.V.); david.girbau@urv.cat (D.G.)

**Keywords:** smart mask, backscatter, double heat flux sensor, temperature, health, LoRa, breathing rate

## Abstract

The COVID-19 pandemic has highlighted a large amount of challenges to address. To combat the spread of the virus, several safety measures, such as wearing face masks, have been taken. Temperature controls at the entrance of public places to prevent the entry of virus carriers have been shown to be inefficient and inaccurate. This paper presents a smart mask that allows to monitor body temperature and breathing rate. Body temperature is measured by a non-invasive dual-heat-flux system, consisting of four sensors separated from each other with an insulating material. Breathing rate is obtained from the temperature changes within the mask, measured with a thermistor located near the nose. The system communicates by means of long-range (LoRa) backscattering, leading to a reduction in average power consumption. It is designed to establish the relative location of the smart mask from the signal received at two LoRa receivers installed inside and outside an access door. Low-cost LoRa transceivers with WiFi capabilities are used in the prototype to collect information and upload it to a server. Accuracy in body temperature measurements is consistent with measurements made with a thermistor located in the armpit. The system allows checking the correct placement of the mask based on the recorded temperatures and the breathing rate measurements. Besides, episodes of cough can be detected by sudden changes in thermistor temperature.

## 1. Introduction

People’s welfare has become the major objective in these times of pandemics. With the arrival of the COVID-19 virus, measures to contain the spread of the disease among the population have been taken. Confinement, use of face masks and gloves, temperature controls at the entrance of public places and social distancing are some of theses measures, which have come to stay for a while.

Authorities have ordered the population to use wearable devices that comply with the quarantine measures, while telecommunication data have been used to monitor crowds. Corporations are working on applications capable of alerting situations in which a person may have come into contact with newly diagnosed COVID-19 patients [1]. For this reason, smart mobile applications have been recently proposed for monitoring purposes, such as employees in industrial environments [2].

However, the pandemic has revealed the limitations of current technological deployments, particularly in what concerns the spread of the disease. IoT and smart connected technologies, in combination with data-driven applications, can play a critical role not only in the prevention, mitigation, and continuous remote monitoring of patients, but also in the prompt enforcement of guidelines, rules, and administrative orders to prevent future outbreaks [1,3]. The pandemic has accelerated the introduction of IoT in several fields such as E-health, smart hospitals, remote patient monitoring, or supply chain management [1,3].

Pandemic has been a race against time, in which the effectiveness of the aforementioned measures has been verified along the way. To avoid a massive contagion in public places, a protocol to measure body temperature has been established, isolating those people with symptoms of COVID, such as fever.

There are several methods for measuring body temperature (e.g., oral, rectal, axillary, or tympanic). Some of these are compared in the literature [4]. Despite the number of options, most of them are not feasible outside of a medical environment and even less on a large scale. Therefore, there is no question why the infrared thermometer was the method of choice for monitoring people’s body temperature. An infrared thermometer allows a quick and non-contact measurement, but the massive use of this kind of thermometer has made visible some latent drawbacks [5]. These include the effect of the distance, the measurement site, or the influence of the environment on the temperature readings. To ensure a correct determination of the temperature, the operators designated for the controls must follow a strict protocol, and anyone who undergoes the measurement requires an acclimatization time when coming from outside. In practice, the system is imprecise and inefficient, despite being the most suitable for mass population control.

Face masks allow the insertion of breathing sensors [6] that allow the person to carry out their usual activity despite the discomfort of their use. The mandatory use of face masks to prevent the spread of coronavirus through the air has led to the adoption of smart masks equipped with sensors. The widespread use of the masks allows us to think of other applications and uses different from those expected in medical or hospital environments [7] for remote monitoring or in industrial environments [8]. Accordingly, a new generation of wearable devices that receives the name of smart masks has attracted the interest of different research groups [7,8,9,10,11,12,13,14,15]. In [7], a personalized smart mask obtained from a 3D scanned face image, whose objective is to minimize skin irritation is proposed. In addition, temperature and strain sensors have been integrated to detect irregular breathing, which is one of the symptoms of respiratory diseases. The strain sensor is used to monitor possible face irritation caused by the tight sealing of the masks. However, the temperature sensor is based on a thermistor connected to a microcontroller that exclusively monitors the air temperature. In [9,12], a smart mask senses particles of different sizes suspended in the air and close to it by using an onboard particulate matter (PM) sensor. Another example of a smart mask is proposed in [10], where a self-powered device based on a textile triboelectric nanogenerator (TENG) is proposed to electrocute virus-loaded aerosols. Recently, in [13] a smart mask with wireless connection using Bluetooth low-energy (BLE) is proposed. It integrates a pulse-oximeter (PPG) and a pair of differential barometers, one internal and one external, that acts as a spirometer and are able to measure respiratory pressure during the breathing cycle.

As in the related works described above, this work takes advantage of the acceptance of the face mask and its widespread use to add a new functionality: it introduces a novel approach to determine the temperature and breathing rate of the person that uses the smart mask. In addition, the solution is capable of granting or denying access to public areas such as academic, leisure, administration, and healthcare centers based on the wireless reading of the sensors placed in the mask, eliminating the error introduced by the operator and improving the screening efficiency. In order to automate the system, the smart mask incorporates a low-power communication system based on the LoRa backscatter technique. Backscatter communication has proven to be of great interest in recent years for the internet of things and power-efficient applications due to the features that this technique provides [16,17,18]. Traditional RF transmitters have a power consumption several orders of magnitude higher than that consumed by backscatter transmitters and, on the contrary, the communication becomes unidirectional. Examples of applications of backscatter communications have been proposed in the literature [19,20,21].

Preliminary results of temperature measurements using a mask with backscatter communication are presentedin [22]. Important limitations of that work are addressed with completely different and more complex solutions here. A new temperature sensor is designed, which does not depend on the person and ambient variations. As COVID-19-compatible symptoms can also manifest in breathing activity, this work also includes breath rate monitoring using an integrated air-flow sensor. Finally, effective location mechanisms are included for motion direction detection, allowing automatic occupancy detection. Other applications of the smart mask proposed in this work, such as monitoring patients while they wait in hospitals or medical facilities avoiding room contamination, could be studied. However, although these applications can be easily derived from the work proposed here, they are out of the scope of this paper.

System design is challenging for at least two reasons: First, determining body temperature through skin temperature is controversial. The face is highly exposed to drafts and sudden changes in ambient temperature, which directly influences the measurements. Second, the backscatter technique makes synchronization between devices difficult due to the one-way communication, and consequently packet collision is a major problem to manage.

Sustainability must be an essential point to consider in any scientific advance. With an estimated monthly use of 129 billion face masks [23], the design of a disposable electronic system integrated into the mask does not appear to be an environmentally friendly solution. The option of integrating the system into a reusable device has been taken into account. Although the electronics do not present disadvantages to be integrated into a dockable prototype, any modification of the mask could affect or invalidate the strict approvals that support it. For this reason, major changes in mask morphology have been avoided. Since this work aims to develop a proof of concept, the discussion of the effect of the system in reference to mask approvals is outside the scope of this paper.

In an effort to solve the aforementioned problems, the main contributions of our work are summarized below:

1. A dual heat flux sensor has been integrated into a commercial FFP2 mask to determine body temperature by using cheek surface temperature.
2. A thermistor has been integrated into the mask to measure the temperature of the air flow, thus being able to monitor both the respiratory rate and the episodes of coughing.
3. Transmission is based on backscatter communication of the signal sent by a LoRa transmitter located next to an access door. The position of the sensor can be determined by comparing the power of the backscattered signal received by two LoRa receivers located on both sides of the access door.
4. The system uses an inexpensive standard LoRa transceiver without any modifications, so no specialized receivers (e.g., software-defined radio) are required.

The paper is organized as follows. Section 2 begins with a brief overview of the design and operation of the prototype as well as the main components of the system. Section 2.2 describes body temperature measurement by means of a dual heat flux sensor. The way to determine the breathing rate using a thermistor that measures the air temperature is explained in Section 2.3. The basis of backscattering communication using LoRa signals is explained in Section 2.4. Experimental results are presented in Section 3. This section starts with the procedure to calibrate the dual heat flow sensor and continues with the results obtained from the measurements of temperature and breathing rate, as well as the detection of cough and positioning. Different non-contact temperature sensor technologies are discussed in Section 4. Finally, the conclusions from this work are summarized in Section 5.

## 2. System Design

### 2.1. System Overview

**Figure 1:**
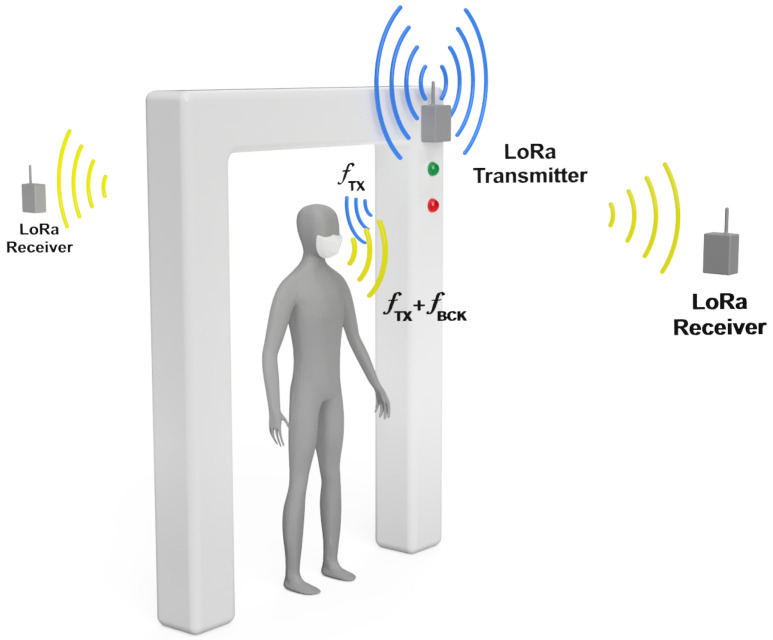
Scheme of the system operation. A LoRa transmitter is placed at the entrance illuminating the subject wearing the smart mask. LoRa receivers placed inside and outside receive the signal backscattered by the mask. Access is granted or denied depending on the value of the temperature readings.

**Figure 2:**
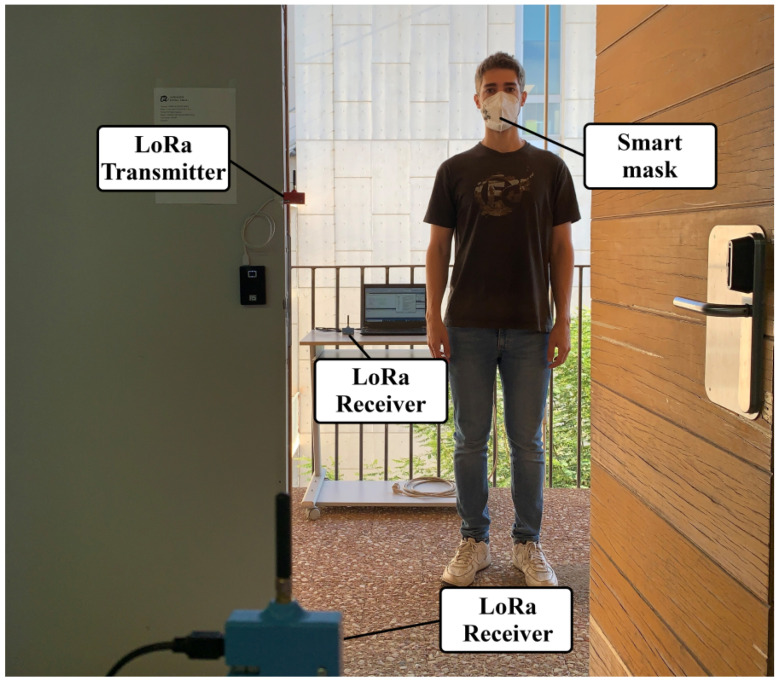
Photography of the measurement scenario. LoRa transmitter is placed at the entrance. LoRa receivers are placed inside and outside of the laboratory.

Figure 1 shows an overall operation sketch of the system. Figure 2 shows a typical scenario where the measurements have been taken. The main goal is to filter people quickly and efficiently without the need for an operator. For this purpose, LoRa transceivers are proposed to be placed at the entrance of public venues. Low-cost LoRa radios ensure scalability and commercialization with a low budget. The transmitter is placed at the entrance, while receivers are placed inside and outside the site. This distribution of LoRa transceivers allows determining the position with a sufficient level of accuracy, by means of the received signal strength. This method is limited by the range of the receiver, few meters around the entrance, but it is sufficient to determine whether or not the subject has crossed the entrance and where it was last detected. This property can be used to implement an automatic occupancy control system. The attenuation of the wall plays an important role in determining if the person is inside or outside. As the temperature sensor is inserted in the mask, it is possible to realize multiple measurements to check the body temperature, unlike when it is done with the infrared thermometer; this allows the system to get a set of temperatures and save them for further evaluation. Receivers work as a gateway, sending the data via WiFi to the cloud or a local server. However, no information that could violate privacy is stored.

**Figure 3:**
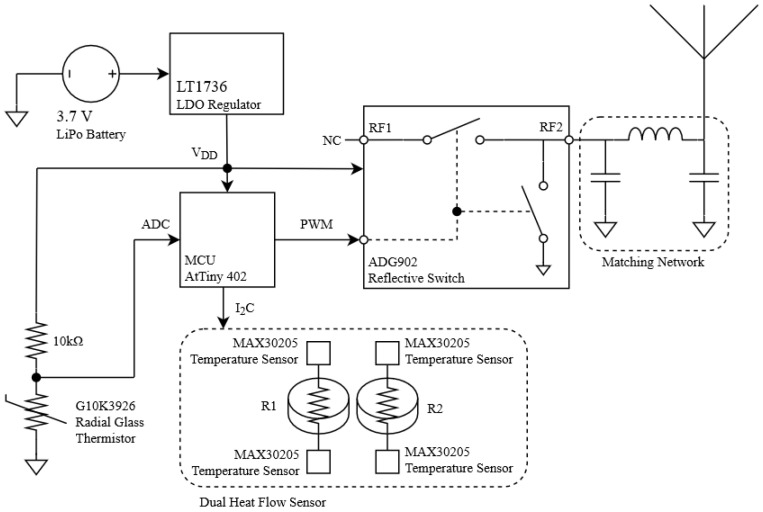
Block diagram of the smart mask.

**Figure 4:**
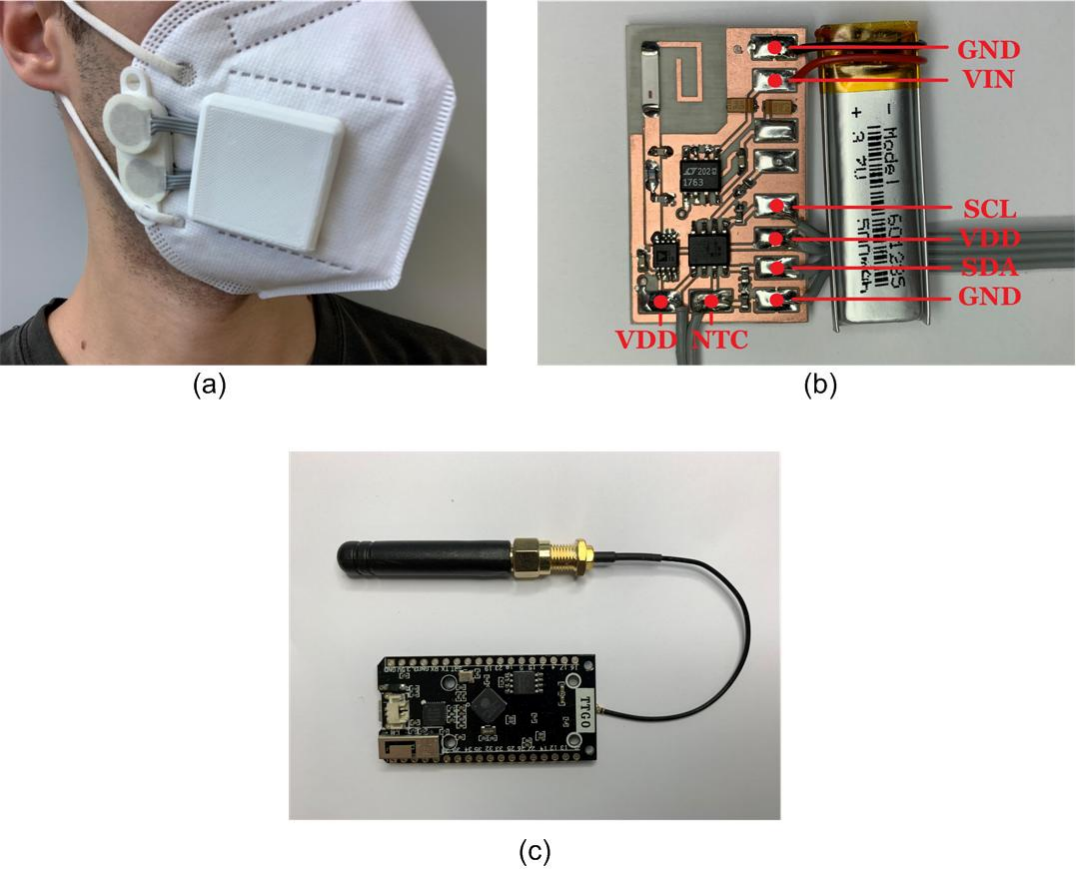
(**a**) Photography of the smart mask. (**b**) Detail of the PCB board. (**c**) ESP32 LoRa transceiver.

A schematic of the transponder placed in the smart mask is presented in Figure 3. It integrates a dual heat flux sensor that determines core body temperature. The high resolution of the integrated circuit used in the design (MAX30205) allows determining, not only the temperature, but also the breathing cycle of the person wearing the smart mask. A low-power microcontroller reads and processes the temperature data. Once processed, data are sent to receivers via LoRa backscatter communication. The system denies access to anyone with a temperature outside the standard ranges, activating an alert and preventing the entrance of the person. When irregular measurements are detected, the mask identifier and temperature data are saved, providing a useful history and allowing the system to record the identifiers of nearby masks to track and be warned of possible infections. Figure 4 shows an image of the prototype coupled to a FFP2 mask. The system is powered by a tiny lithium polymer battery with a capacity of 500 mAh.

The theoretical basis that supports the design of the system can be divided into three main parts. The first subsection aims to study the measurement of body temperature using a dual-heat-flux sensor installed in the mask. The second subsection addresses the measurement of breathing rate from an airflow sensor. Finally, the third part describes the LoRa backscatter communication.

### 2.2. Temperature Sensing

Determining body temperature through the skin has always been a challenge [24]. Although conventional thermometers can perform this task precisely, the locations to measure the temperature must be very sheltered. The most common measurement places are the oral, tympanic, axillary, and rectal. All of them are unfeasible for measuring temperature on a large scale or outside of a healthcare environment [25]. Infrared thermometers have been the only method capable of handling the pandemic situation due to a quick and non-intrusive way of measuring body temperature, but their accuracy and reliability have been questioned in practice [26,27,28,29].

The simplest way to measure body temperature is by measuring skin temperature with a temperature sensor (e.g., with a thermistor or an active integrated circuit sensor). Although this method works well in places where the temperature is stable (e.g., in the armpit), it is not the most ideal system to be integrated into a mask, as it is highly exposed to drafts and changes in temperature. A method for determining deep body temperature is through heat flux sensors. Several works that analyze the performance of this kind of sensors can be found in the literature [30,31,32]. In [30], a single heat flux sensor based on temperature measurement at two points plus a correction to take into account the influence of ambient temperature is proposed. However, the calibration of the sensors depends on the thermal conductivity of the body. To solve this problem, dual heat flux sensors can be used to measure the temperature in four points [31].

**Figure 5:**
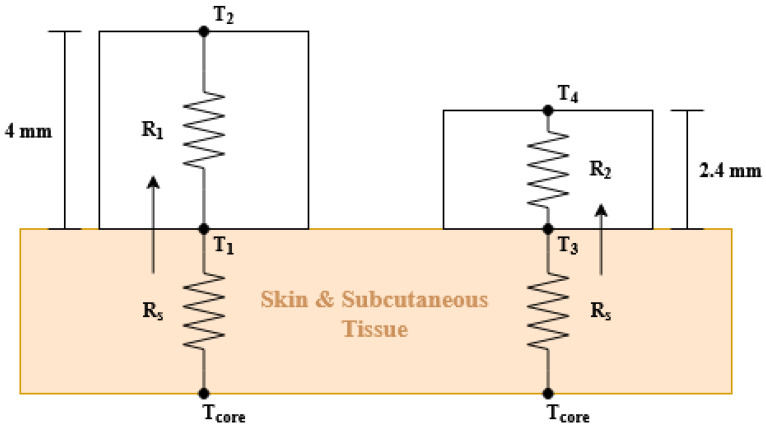
Schematic diagram of the dual heat flux sensor and its equivalent circuit.

In a previous work [22], two thermistors were used to estimate the body temperature: one installed in the cheekbone and the other outside the mask to measure ambient temperature. With this methodology, it is necessary to calibrate the parameters of the model for each person because they depend on the skin resistance. In addition, the thermistor must be calibrated with great precision. To avoid these problems, in this work, a dual heat flux (DHF) probe is designed and embedded into the face mask. Figure 5 shows the operating diagram of the core temperature probe. It consists of two heat flow sensors integrating four MAX30205 temperature sensors from Maxim Integrated, one MAX30205 pair for each heat flux sensor. This chip has an accuracy of 0.1 °C and is specifically designed to measure the temperature of the human body. Both MAX30205 are isolated from each other with polylactic acid (PLA), which has a thermal conductivity equal to 0.13 W/m · K.

According to the second law of thermodynamics, heat will flow from the hottest point to the coolest point until both temperatures are equalized by diffusion. The dual heat flux probe can be modeled with an electrical system [31,32] described in Figure 5. In this analogy, temperatures are represented by the voltages in the nodes and the heat flow is the current that flows between two nodes. Analyzing this circuit, the following equations are obtained:

(1) $$T_{core}=T_{1}+\frac{\left(T_{1}-T_{2}\right)R_{S}}{R_{1}}$$

(2) $$T_{core}=T_{3}+\frac{\left(T_{3}-T_{4}\right)R_{S}}{R_{2}}$$

where $T_{1}$ and $T_{3}$ are the temperatures on the skin surface, $T_{2}$ and $T_{4}$ are the temperatures at the top surface of each heat flux sensor, $R_{S}$ is the thermal resistance of the skin and the subcutaneous tissue, and they have the same value for the two flux sensors. That is a reasonable approximation, considering that both sensors are very close to each other. The problem arises because the thermal resistance $R_{S}$ of the tissue beneath each heat flux sensor cannot be measured and it is strongly influenced by the hypodermic blood flow and subject to variations between persons. The use of two heat flow sensors solves this problem. Eliminating $R_{S}$ from (1) and (2), the core temperature can be obtained from the measured temperatures:

(3) $$T_{core}=T_{1}+\frac{\left(T_{1}-T_{2}\right)\left(T_{1}-T_{3}\right)}{K\left(T_{3}-T_{4}\right)-\left(T_{1}-T_{3}\right)}$$

where *K* is defined as the ratio of thermal resistances $R_{1}$ and $R_{2}$ ($K=R_{1}/R_{2}$). The ratio *K* is determined during the calibration procedure from a known $T_{core}$ and the measured temperatures:

(4) $$K=\frac{\left(T_{core}-T_{3}\right)\left(T_{1}-T_{2}\right)}{\left(T_{core}-T_{1}\right)\left(T_{3}-T_{4}\right)}$$

**Figure 6:**
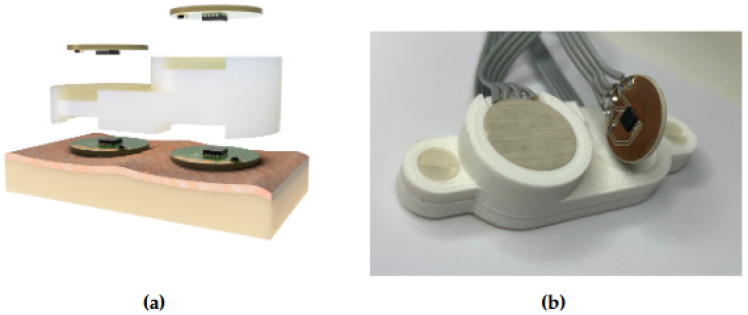
Dual heat flow sensor prototype built on a 3D printed PLA support, which acts as an insulator between the four temperature sensors: (**a**) 3D rendering of the prototype and (**b**) real prototype.

In the implemented prototype, a PLA housing that integrates and isolates the four sensors has been printed with a 3D printer, as can be observed in Figure 6. The height of each heat flux sensor is 5 mm and 10 mm, respectively, and the diameters are 20 mm. The body temperature $T_{core}$ can be calculated after estimating the ratio *K* using (3). Experimental results will be provided in Section 3.

### 2.3. Breathing Monitoring

COVID-19 disease can cause shortness of breath, lung damage, and impaired respiratory function [33]. COVID-19 patients can develop a complication known as Acute Respiratory Distress Syndrome (ARDS). With ARDS, patients lose the ability to breath normally, and this is known as a respiratory failure that results from severe inflammation in the lungs. Therefore, anomalies in breathing rate can be useful for screening COVID-infected subjects. In addition, an increase in temperature is observed in people after having done some physical exercise [34]. Therefore, tracking the breathing rate history can be useful in avoiding false positives.

**Figure 7:**
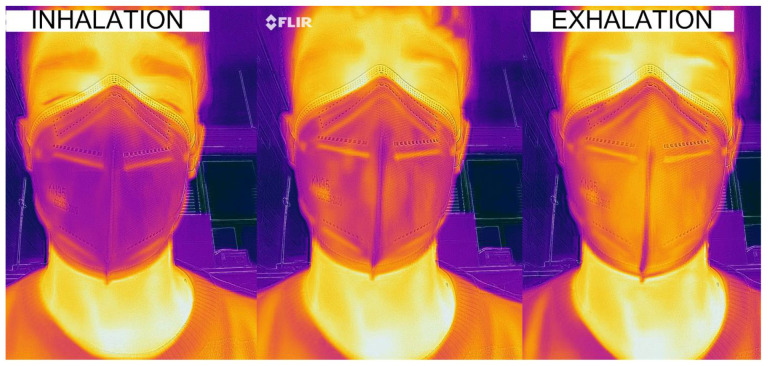
Thermal image of a breathing cycle of a subject with a mask.

Figure 7 shows the thermal behavior inside the mask (FFP2 type) during a respiration cycle. The temperature in the cheekbone and the lateral part of the mask remains mainly constant, whereas the central part of the mask changes according to the inhaled and exhaled air temperature. Therefore, the dual heat flux sensor (DHF) must be installed on the lateral part of the mask to avoid heating associated with breathing. To monitor breathing, a temperature airflow sensor capable of detecting temperature variations is used. The temperature airflow sensor consists of a Negative Temperature Coefficient (NTC) thermistor integrated into the mask and located under the nose. Compared to other temperature sensors, NTC devices are inexpensive and are characterized by both high sensitivity and high nonlinearity. In this design, absolute temperature is not required. Consequently, the calibration and the nonlinearity of this sensor are not critical issues. In the manufactured prototype, a radial glass NTC model G10K3976 from TE Connectivity is employed.

The first-order approximation of the Steinhart–Hart equation [35] is given by the manufacturer to model the NTC resistance *R* as a function of the temperature:

(5) $$\frac{1}{T}=\frac{1}{T_{0}}+\frac{1}{\beta }ln\left(\frac{R}{R_{0}}\right)$$

where *T* is the temperature in K, $R_{0}=$ 10 kΩ is the nominal resistance at $T_{0}$ = 298 K, and β = 3976 K is the Steinhart–Hart parameter, which is provided in the thermistor datasheet.

A simple voltage divider composed of the NTC and a resistance with the nominal value of the NTC (10 kΩ) connected to the supply voltage $V_{cc}$ is used to obtain the breathing measurement. The voltage output $V_{out}$ of this circuit is acquired with the internal analog-to-digital converter (ADC) of the microcontroller:

(6) $$V_{out}=V_{cc}\frac{R}{R+R_{0}}$$

In order to estimate the limitation in the temperature measurement due to the finite resolution of the ADC, the voltage sensitivity can be obtained combining (5) and (6) and derive the result with respect to the temperature:

(7) $$\frac{dV_{out}}{dT}=\frac{dV_{out}}{dR}\frac{dR}{dT}=-V_{cc}\frac{R\cdot R_{0}}{{\left(R+R_{0}\right)}^{2}}\frac{\beta }{T^{2}}\approx -\frac{V_{cc}}{4}\frac{\beta }{T^{2}}$$

For temperatures in the order of ambient temperature, $T\approx T_{0}$ gives an approximate value of −35 mV/K. Taking into account that the temperature variation due to airflow is on the order of 4 to 5 degrees, the microcontroller’s 10-bit ADC, provides enough resolution to digitize the output without the need for amplification, thus reducing the number of components.

This temperature signal estimated from the airflow sensor has a small variation over the average temperature of the mask and it is also noisy. Therefore, after the average signal is subtracted, low-pass filtering is applied to reduce noise. As the filters must be implemented in a low-power microcontroller, exponential moving average (EMA) filters are used to reduce the computational charge and memory. The average temperature $X_{av}\left[n\right]$ is estimated using the following recursive equations from the measured temperature samples X[n] [36]:

(8) $$X_{av}\left[n\right]=\alpha_{1}X\left[n\right]+\left(1-\alpha_{1}\right)X_{av}\left[n-1\right]$$

The output breathing signal Y[n] is obtained from a second exponential moving average filter applied to X[n] after subtracting the average value to reduce noise:

(9) $$Y\left[n\right]=\alpha_{2}\left(X\left[n\right]-X_{av}\left[n\right]\right)+\left(1-\alpha_{2}\right)Y\left[n-1\right]$$

Typical values for $\alpha_{1}$ and $\alpha_{2}$ are 0.04 and 0.08, respectively.

The breathing rate is usually expressed in breaths per minute (bpm). It can be estimated from the inverse of the interval between two consecutive peaks of the breathing signal. A robust peak detection algorithm described in [37] is applied to the filtered breathing signal Y[n]. When an interval between two consecutive breathings is longer than 10 s, it is considered as apnea. In this case, an apnea index is activated to count the number of apneas during the measurement session.

Compared to conventional airflow sensors used for breath monitoring, the integration of the airflow sensor in the mask allows for comfortable breath monitoring once the obligation to wear the mask is assumed.

### 2.4. Tag Detection

**Figure 8:**
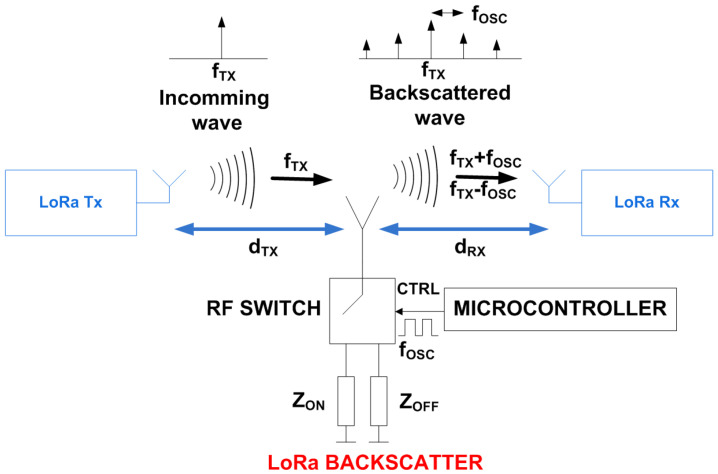
Block diagram of a LoRa backscatter.

**Figure 9:**
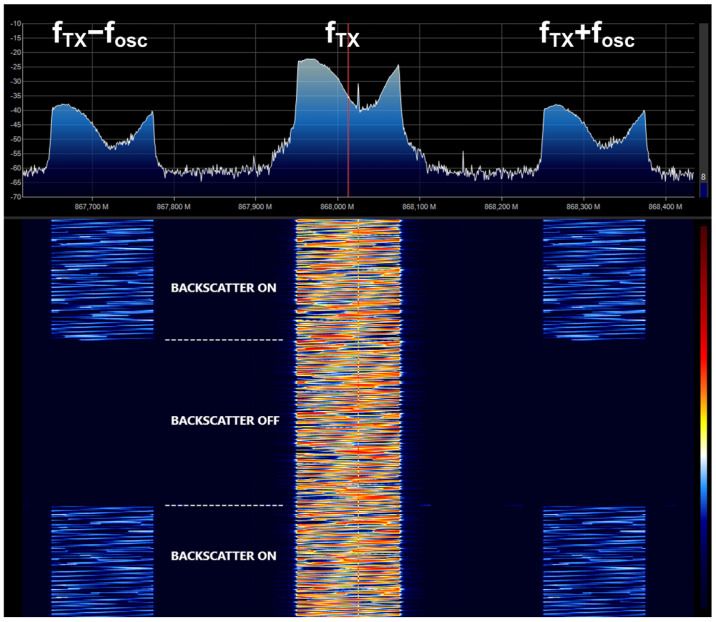
Measured spectrogram with backscatter on and off showing the shift of LoRa channel to offset channels when backscatter is on.

The feasibility of using backscatter communication for tag positioning and data communications is explored in this section. Backscatter communication is based on the principle of load modulation, where the impedance that is connected to the antenna is switched between two states. The result is a modulation of the incoming waves without creating its own RF transmission signal (see the schema of Figure 8). The switch works as a mixer. Consequently, the spectrum of the backscattered signal presents a shift of $\pm nf_{osc}$ respect to the frequency of the incoming signal $f_{TX}$, where n is an integer that indicates the harmonic index. The amplitudes of these harmonic components are proportional to the Fourier coefficient of the waveform that controls the switch (see in [21] for details). Therefore, the amplitudes of the highest-order harmonics decrease very quickly, placing them below the noise floor and making them very difficult to detect. Different RF sources can be used such CW tones, WiFi, Bluetooth, or, as in this case, LoRa signals. In this work, the backscatter communication works over standard LoRa modulated packets. LoRa is a spread spectrum modulation derived from the existing Chirp Spread Spectrum (CSS) technology. High receiver sensitivity and robustness in front of interferences make LoRa ideal for low-power, long-range, and low data rate applications [21,38,39]. If the oscillator frequency is properly selected, the signal reflected at the tag contains information on the adjacent channels. Therefore, when the backscatter has the modulation enabled, LoRa receivers tuned at the frequency of the shifted channel $f_{TX}\pm nf_{osc}$ can demodulate packets sent by a LoRa transmitter tuned at $f_{TX}$. Therefore, data can be sent at low speed by connecting and disconnecting the oscillator of the backscatter, observing the presence of the backscatter information in the shifted channels. Figure 9 shows the measured spectrogram (spectrum as a function of the acquisition time) at the output of an antenna connected to a software-defined radio receiver (RTL-SDR). This figure shows signal reception on the shifted channels when the backscatter modulation is enabled.

The backscattered received power ($P_{R}$) at the shifted backscatter channel (at frequency $f_{TX}\pm nf_{osc}$) located a distance $d_{R}$ from the transmitter can be estimated as in a conventional RFID system using the radar equation:

(10) $$P_{R}=\frac{P_{T}G_{T}}{4\pi d_{T}^{2}}RCS_{dif}\frac{1}{4\pi d_{R}^{2}}\frac{\lambda^{2}}{4\pi }G_{R}$$

where $P_{T}$ is the LORA transmitted power, $G_{T}$ the transmitter antenna gain, $G_{R}$ is the receiver antenna gain, $RCS_{dif}$ is the differential radar cross section of the backscatter, λ is the wavelength, and $d_{T}$ and $d_{R}$ are the distances from the transmitter to the backscatter and from the backscatter to the receiver, respectively.

Therefore, the modulated backscatter power is a function of the differential radar cross section $RCS_{dif}$ that depends on the tag parameters such as the tag antenna gain, the mismatch between the two impedance states [40], and the modulation waveform:

(11) $$RCS_{dif}=\frac{\lambda^{2}}{4\pi }G_{a}^{2}|\Delta \Gamma {|}^{2}m$$

where $G_{a}$ is the gain of the antenna, $\Delta \Gamma =\Gamma_{ON}-\Gamma_{OFF}$ is the difference between the complex reflection coefficients of each state, and *m* is the modulating factor, equal to $\frac{1}{\pi^{2}}$ considering an ideal 50% duty cycle and a square wave modulation. The complex reflection coefficients $\Gamma_{i}$ can be computed from the antenna impedance $Z_{a}$ and the load impedance of each state $Z_{i}$ (*i* = ON, OFF):

(12) $$\Gamma_{i}=\frac{Z_{i}-Z_{a}^{*}}{Z_{i}+Z_{a}}$$

The mask prototype uses an AtTiny402 microcontroller powered by a 500 mAh lithium polymer (LiPo) battery. The microcontroller reads the temperature, processes the information, and generates the square wave signal to control the RF switch. The power consumption of the microcontroller and the RF switch together does not exceed 620 μA. The critical part regarding power consumption lies in the DHF probe, which uses four MAX30205 ICs with a supply current of 600 μA each one. Therefore, the maximum power of the system can reach 2.8 mA. In the worst-case scenario, with all components running at 100% duty cycle, the battery life could go up to 179 h. Considering that MAX30205 has a shutdown mode with a current draw of less than 3.5 μA, and that it only takes 50 ms to perform the temperature conversions, the total power consumption of the system can be greatly reduced by adjusting the duty cycle of the DHF sensor. The switch chosen for this application is an ADG902 from Analog Devices. Attached to the switch there is a 0868AT43A0020 ceramic antenna from Johanson Technology matched with an LC network.

**Figure 10:**
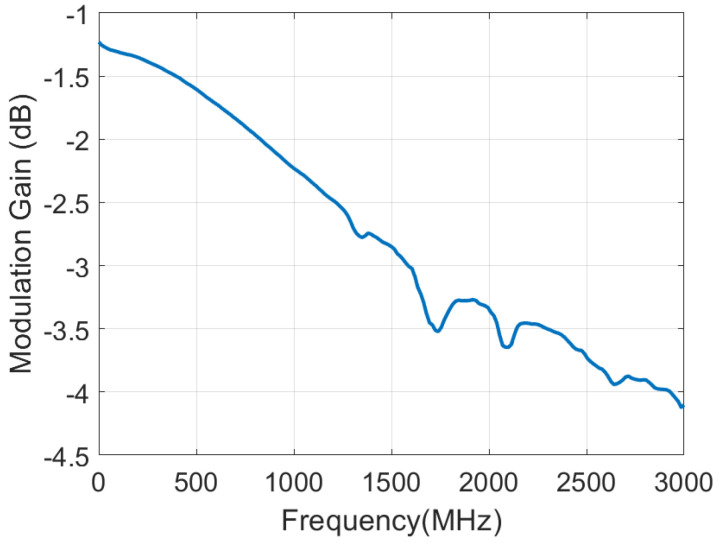
Measured modulation gain as a function of the frequency for the ADG902 switch.

Figure 10 shows the measured modulation gain defined as the ratio between the backscattered power of the switch and the backscattered power of an ideal switch loaded with open-circuit and short-circuit (${\left|\Delta \Gamma \right|}^{2}/4$). It has been obtained measuring the reflection coefficient at the antenna port using a VNA and switching between an open and a short circuit connected at the input of the ADG902, previously mounted in a test PCB accessible with a SMA connector. The modulation factor decreases with frequency due to increasing switch losses. The modulation factor is approximately −2 dB for an 868 MHz ISM band, which is an acceptable value for this application.

To determine if the smart mask is inside or outside of the building or room, two LoRa receivers are installed at the entrance of a building or room, as it is shown in Figure 3, one inside and one outside. Both receivers are tuned at a frequency-shifted channel. The transmitter is installed at the entrance, right in the middle point. The distance between the receivers and the transmitter is set to 2 m in the experiments. The use of two receivers has two main advantages, such as increasing the probability of receiving data packets under non-line-of-sight (NLOS) propagation conditions and determining if the subject has entered the building by comparing the received signal strength intensity (RSSI) of each receiver. From (10), the received power should be higher for the receiver closer to the tag and therefore it is possible to know if the person has crossed the door and in which direction. A simple classifier can be established from the RSSI or the signal-to-noise ratio (SNR) measured by the commercial LoRa receiver for the received packets. Unlike other backscatter-based communication systems proposed in the literature that require specific and often expensive SDR receivers, the system proposed here allows the use of commercial devices without having to make any modification. The system has been implemented using commercial Semtech SX1276 LoRa Transceivers. A low-cost (about 15 €) module from TTGO that integrates an ESP32 microcontroller with WiFi and a Semtech SX1276 is used in the experiments. LoRa transceivers are set with a spreading factor (SF) of 7, a bandwidth of 250 kHz, and a preamble length of 6. The air time of the packets is 18 ms. LoRa transmitter frequency is set to 868 MHz and backscatter shifting frequency $f_{osc}$ is set to 500 kHz. Consequently, LoRa receiver’s frequencies are set to 868.5 MHz. The power of the transmitter is set to 17 dBm.

**Figure 11:**
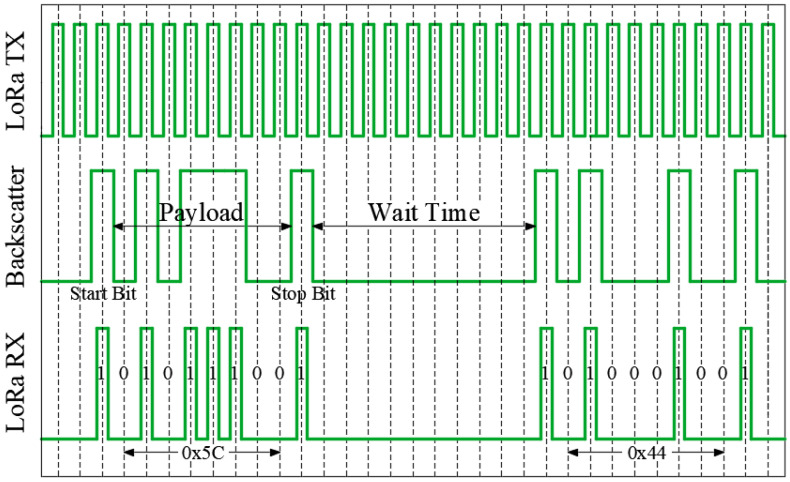
Backscatter communication protocol diagram.

An on–off keying (OOK) amplitude modulation has been implemented in the smart mask (see Figure 11). The LoRa transmitter sends packets sequentially spaced 14 ms between them. When backscatter is activated, the LoRa packets are bounced to the side channel where the receivers are listening. The backscatter communication protocol sends a byte of data and two start/stop bits to indicate the start and the stop of the payload. Therefore, the frame length is 10 bits. The duration of each bit (32 ms) is the sum of a LoRa packet and the gap between each one of them. As the backscatter leaves a minimum time between frames equal to its frame length and considering the values mentioned above, the system can send frames every 640 ms, therefore the data rate is 12.5 bits/s. The package payload includes two main parts, the identifier and the sensor data (e.g., temperature or breathing rate). The identifier is vital in determining which data comes from each mask, taking the ones with a valid identifier and rejecting the ones that have crashed.

To avoid packet loss due to packet collisions between multiple smart masks, anti-collision strategies have been implemented. The first one is to set properly the LoRa parameters to reduce the communication range, capturing only the signal from the masks near the entrance. The second one consists of randomly adjust the waiting time between frame transmissions, thus avoiding potential collisions between frames from different backscatters.

Several results on determining the relative position of the smart mask when walking through the door will be presented in Section 3.3.

## 3. Results

### 3.1. Calibration of the Temperature Sensor

**Figure 12:**
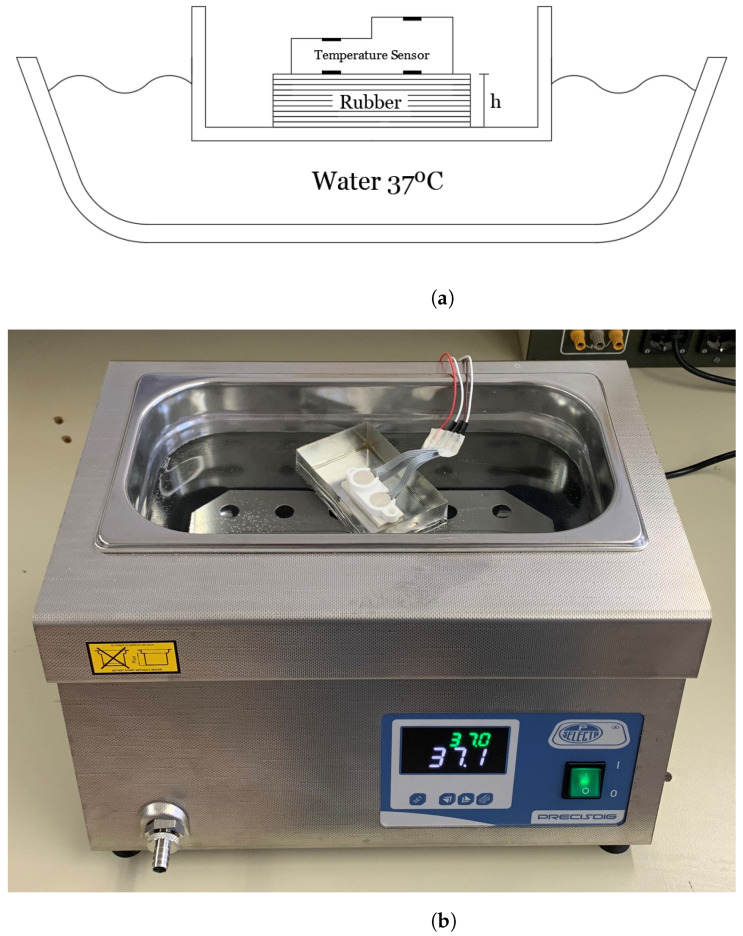
Scheme of the body simulation method used to calibrate the DHF sensor (**a**) and photography of the experimental equipment used to calibrate the DHF sensor (**b**).

**Figure 13:**
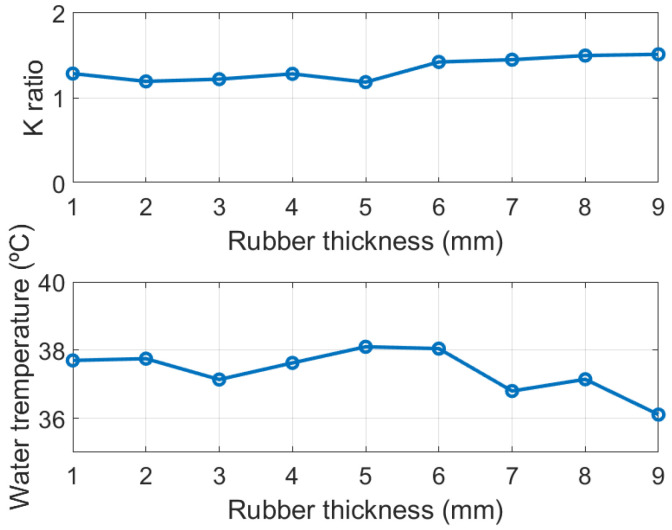
Thermal resistance ratio K (**top**) and estimated water temperature (**bottom**) as a function of rubber thickness.

In order to find the thermal resistance ratio *K* an experimental setup similar to the one proposed in [41] is conducted (see schema in Figure 12). This experiment allows to set the core temperature and check the calibration. A water bath at a temperature of 37° simulates the nominal temperature of the body. A copper container is placed floating on the water. Rubber sheets were used to emulate the skin and subcutaneous tissue. The thickness of the rubber can be increased by adding more sheets. Rubber material has been chosen because its thermal conductivity (0.17 Wm${}^{-1}$·K${}^{-1}$) is close to the value of the subcutaneous tissue. Then, the probe is located on the rubber layer and the thermal resistance ratio *K* is determined for different thicknesses from the measurement of the four temperature sensors using (4). The water temperature is monitored with a thermometer and controlled with a hot plate (with resolution of ±1 °C). A nearly constant value as a function of the thickness of the rubber layer has been obtained (see the top plot in Figure 13). The room temperature is 25 °C. Using the value of the ratio K=1.2 that has been found, the water temperature is estimated from another set of measurements (Figure 13). The differences produced could be attributed to poor lateral insulation of the prototype [32]. Despite this drawback, the error is typically below 0.5 °C.

**Figure 14:**
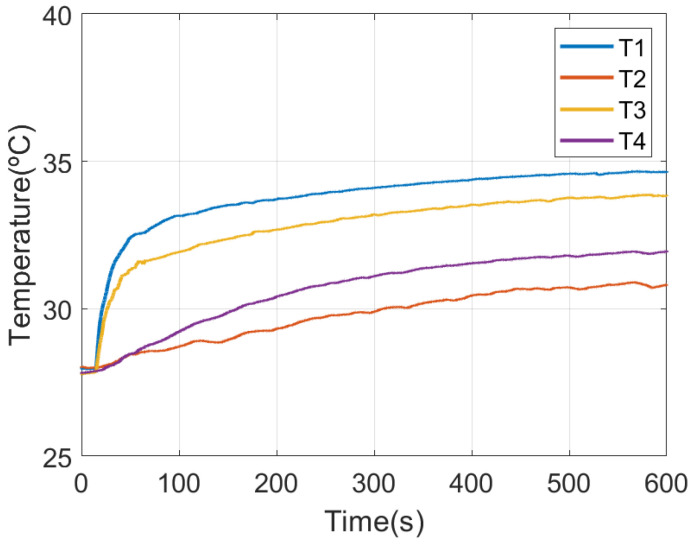
Measured temperature for each temperature sensor in the probe as function of the time after installing the smart mask.

**Figure 15:**
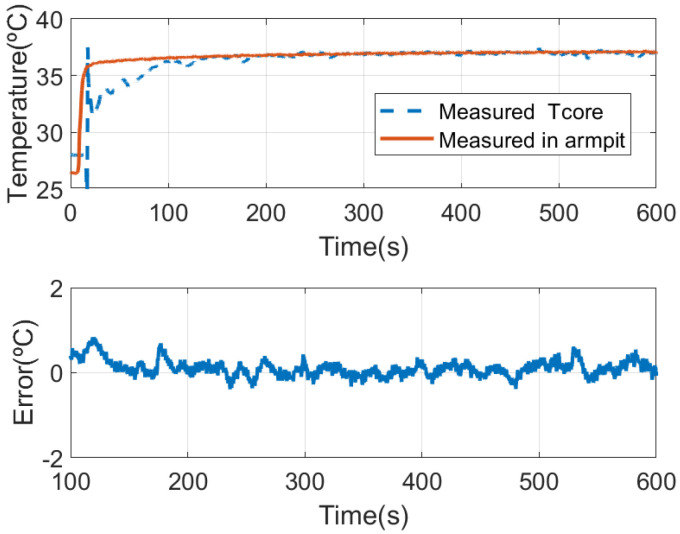
Comparison of the estimated core temperature with the dual heat flux sensor and measured in the armpit with a thermistor (**top**) and difference between both measurements after the initial response (**bottom**).

From the thermal resistance ratio *K* found with the latest experimental setup, the core temperature of a subject wearing the face mask can be estimated from (3). A set of measurements is shown in the following figures. The experiments have been repeated with different persons and similar conclusions have been achieved. The measured temperatures for each sensor of the DHF sensor, following the notation of Figure 5, are shown in Figure 14. The estimated core temperature and the measured temperature in the armpit with a thermistor are shown in Figure 15. It takes ~100 s to reach a value close to the core temperature. Figure 15 shows the difference between two sensors once the temperatures reach the thermal equilibrium. The standard deviation error is 0.2 °C which is of the order of the temperature sensor accuracy. When the mask is not worn, the four temperature sensors tend to ambient temperature, and therefore it is useful to verify the correct placement of the face mask.

### 3.2. Determination of Breathing Rate and Coughing Events

**Figure 16:**
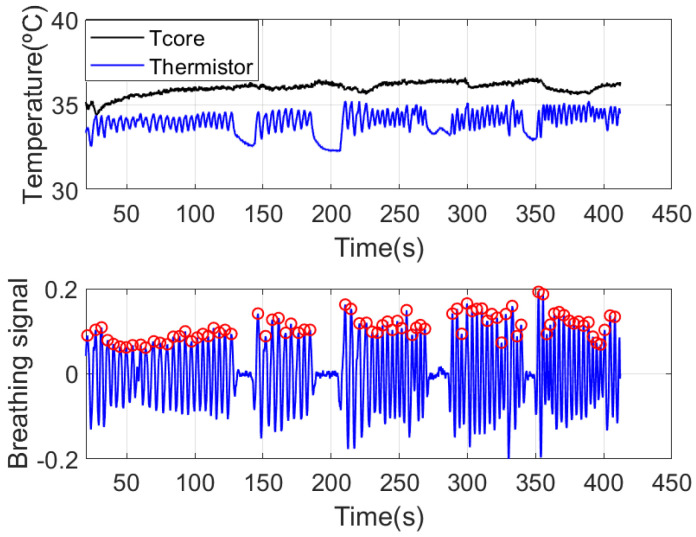
Estimated core temperature and measured frontal thermistor temperature for breathing rate estimation (**top**). Breathing signal after removing and filtering the baseline (**bottom**).

**Figure 17:**
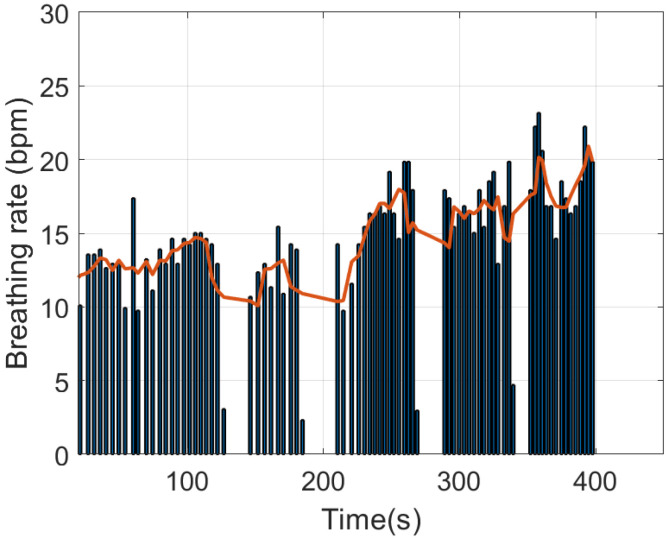
Estimated breathing rate (vertical bars) and average breathing rate (red solid line).

An example of breathing rate estimation is shown in the following figures. Figure 16 shows the measured temperature by the frontal thermistor installed in the mask close to the nose area, in addition to the core temperature estimated with the DHF probe. Some apnea periods have been simulated by consciously stopping breathing. The maximum and minimum peaks obtained with the peak search algorithm are also plotted on the graph. The breath frequency can be obtained from the time position of the maximum peaks (see Figure 17). The average breathing is also shown in this figure. Apneas can be identified if the time interval between two consecutive breaths is greater than 10 s. An increase in breathing amplitude is observed after the end of each apnea period. The breath frequency can be obtained from the time position of the maximum peaks (see Figure 17). The average breathing is also shown in this figure. Apneas can be identified if the time interval between two consecutive breaths is greater than 10 s. An increase in breathing amplitude is observed after the end of each apnea period. These figures show how the smart mask can be used to monitor breathing activity. Some simple statistics such as the average breathing rate, the maximum breathing rate, and the apnea count can be saved in the flash memory of the microcontroller. For continuous data logging, an SD memory card can be used to store the complete data over time.

**Figure 18:**
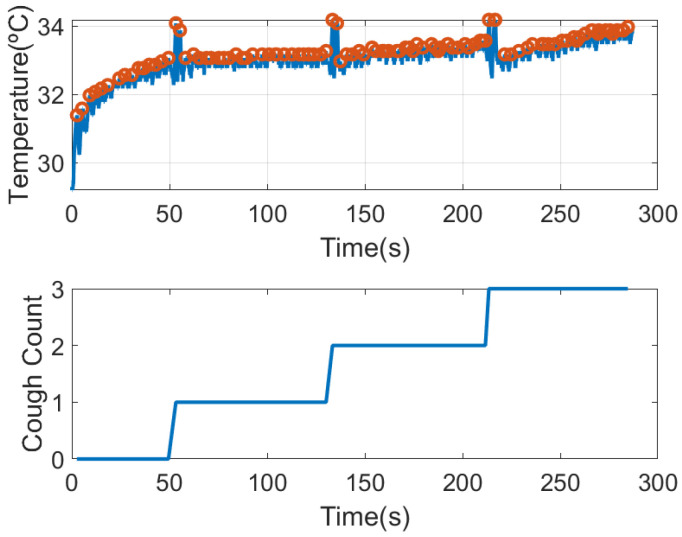
Thermistor temperature measured after putting on the mask considering coughing episodes (**top**). Number of cough episodes detected from maximum temperature peaks (**bottom**).

The presence of cough is another symptom associated with COVID. The system has the ability to detect cough events. Figure 18 shows the temperature of the thermistor after installing the mask. It can be shown that it takes about 25 s to reach the steady state. The figure shows the maximum peaks associated with the breaths. Under these conditions, the amplitude of the peaks remain nearly constant. To show the detection of cough, in this figure, the subject has coughed three times. Coughs cause a rapid rise in temperature of ~1 degree due to the sudden expulsion of air. Therefore, by detecting these peaks and comparing the level with the average of the last 5 temperature peaks, a simple cough counter can be implemented (see graph at the bottom of Figure 18).

### 3.3. Wireless Measurement and Positioning

In [20], a wide-range localization method using LoRa backscatters based on a fingerprint method and machine learning classifiers is proposed. The first requirement of the system is the installation of LoRa receivers distributed in each room. Additionally, a calibration of the classifier is required to obtain accurate location results depending on the fingerprint method used.

**Figure 19:**
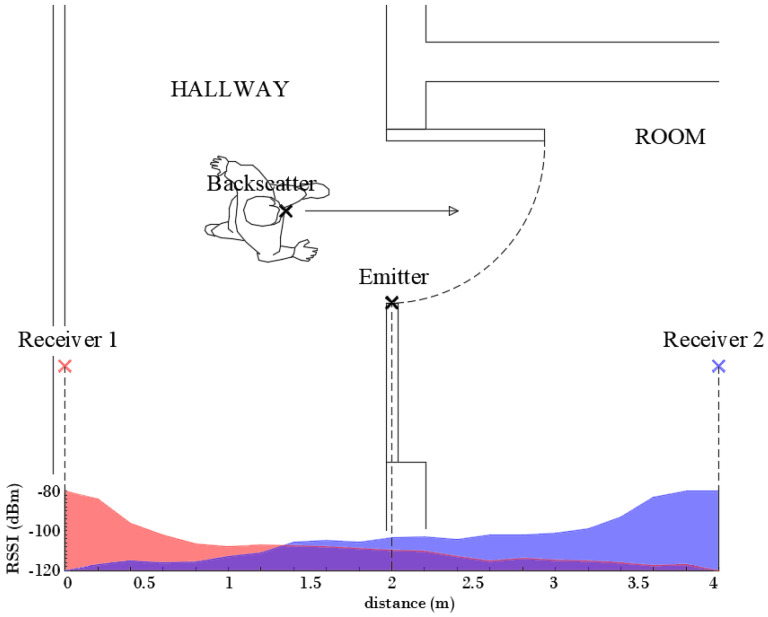
Measured RSSI at the two receivers (receiver 1 outdoor and receiver 2 indoor) for a subject crossing a door, as a function of distance.

The work herein focuses on knowing the relative position of the person with respect to the door, establishing if the person is inside or outside the room. Therefore, a simpler classifier that does not require a training procedure is investigated. Some tests have also been done to evaluate the feasibility of locating the person from the backscattered signal using the setup shown in Figure 19, which corresponds to a typical access control (see Figure 2). LoRa ICs are equipped with measured received signal strength intensity (RSSI) and SNR indicators. The values of these measured parameters are stored in the corresponding registers and can be read via the SPI bus by the host processor. Figure 19 shows the decrease of the RSSI at receiver number one (placed outside) and the increase at receiver number two (placed inside) as the person walks through the door from outside to inside.

However, from (10) the received power would be expected to increase significantly as the person gets closer to the transmitter. Although there is a slight increase, it was expected to be larger. This is attributed to both diffraction effects and the blocking of the direct line of sight produced by the walls and the body itself. The measured RSSI for each receiver presents the crossover at approximately the equidistant point between each receiver and the transmitter. Following (10), the power at the receivers should be equal for equal distances between the transmitter and each receiver. This defines a plane located midway between both receivers. However, due to the attenuation of the walls, slight variations in the positioning of the receiver and, in general, a non-symmetrical scenario, a displacement of the crossover point may occur. A threshold difference parameter (ΔRSSI) to establish the crossover point at the door entrance can be considered. The smart mask will be located inside when the difference between the RSSI measured at each receiver is greater than this parameter ΔRSSI:

(13) $$RSSI_{2}-RSSI_{1}>\Delta RSSI$$

This parameter can be determined experimentally when the receivers are installed. The backscatter is placed in the center of the door and then the difference of the measured RSSI values of the two receivers is averaged. An analog decision rule can be applied using the SNR measurements returned by the transceiver.

**Figure 20:**
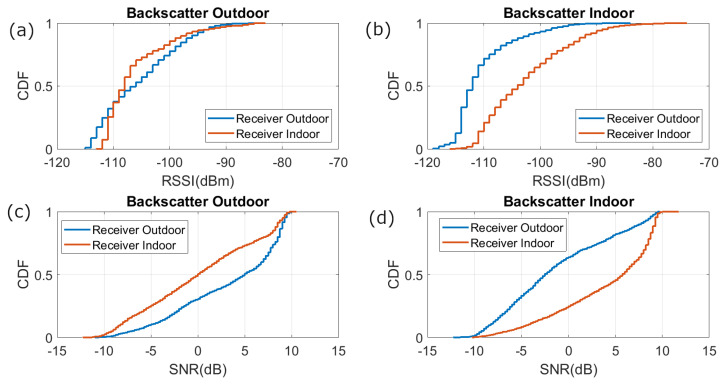
Cumulative distribution function of the RSSI for (**a**) backscatter outdoor and (**b**) backscatter indoor, and SNR for (**c**) backscatter outdoor and (**d**) backscatter indoor.

To study the reception coverage, several measurements have been made with the backscatter device within a radius of three meters around the entrance. Figure 20 shows the cumulative distribution function (CDF) of the RSSI and the signal to noise ratio (SNR) received for points randomly taken both outside and inside the room. These figures show that the measured RSSI and SNR are higher for the receiver closest to the mask. The average SNR returned by the LoRa transceiver has higher resolution than RSSI, so that it is preferred to use the measured SNR values than RSSI values. Therefore, a simple classifier based on the measured SNR can be used to determine whether the smart mask is inside or outside the building or room. The smart mask is outside if the SNR of receiver 1 (at the outdoor) is higher than that of receiver 2 (at the indoor). The read range can be increased by varying the LoRa parameters and the power of the transceivers. The LoRa transceivers are connected to internet, and the information gathered is sent by Message Queueing Telemetry Transport (MQTT) protocol to a broker that can be accessed from any web browser or mobile app.

**Figure 21:**
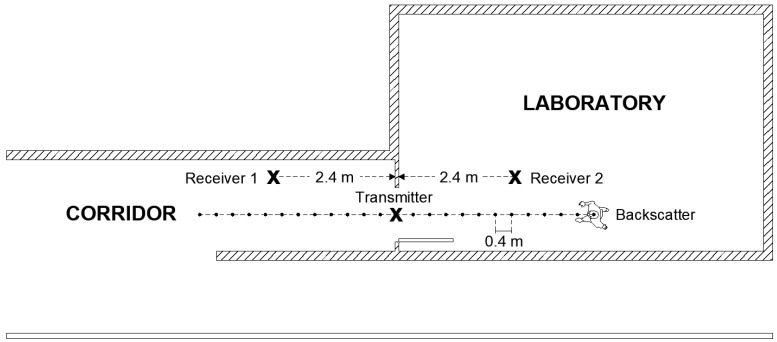
Diagram of the scenario used in the measurements.

**Figure 22:**
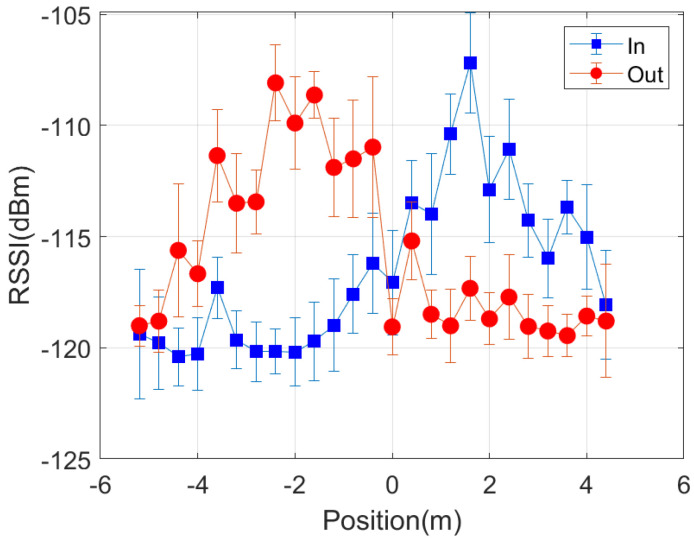
Average RSSI measured as a function of the backscatter position with respect to each of the receivers. The standard deviation has been included in the error bars.

**Figure 23:**
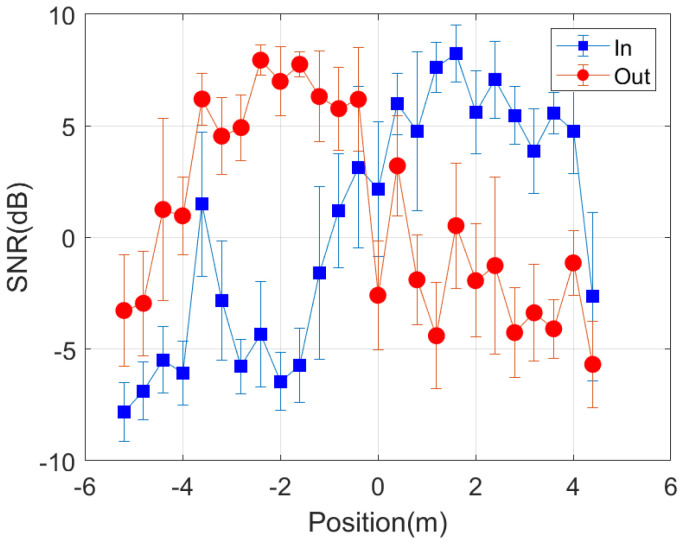
Average SNR measured as a function of the backscatter position with respect to each of the receivers. The standard deviation has been included in the error bars.

**Figure 24:**
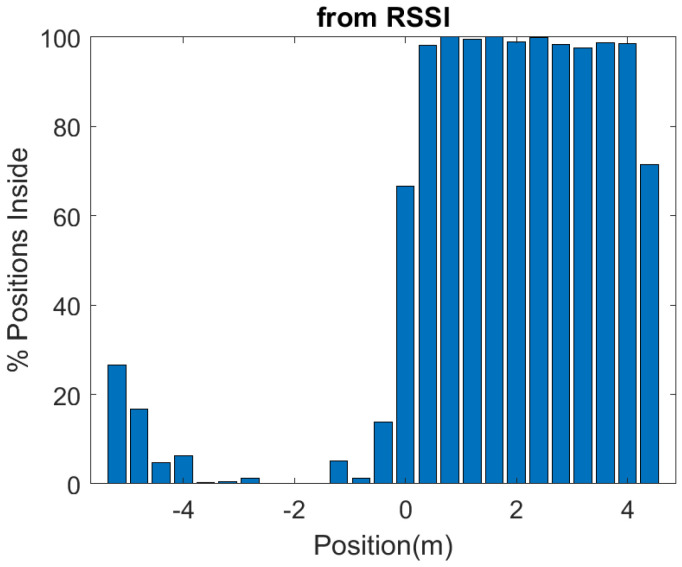
Percentage of cases in which the smart mask is classified inside, as a function of the position with respect to each of the receivers, obtained by comparing the measured RSSI of each receiver at each position.

**Figure 25:**
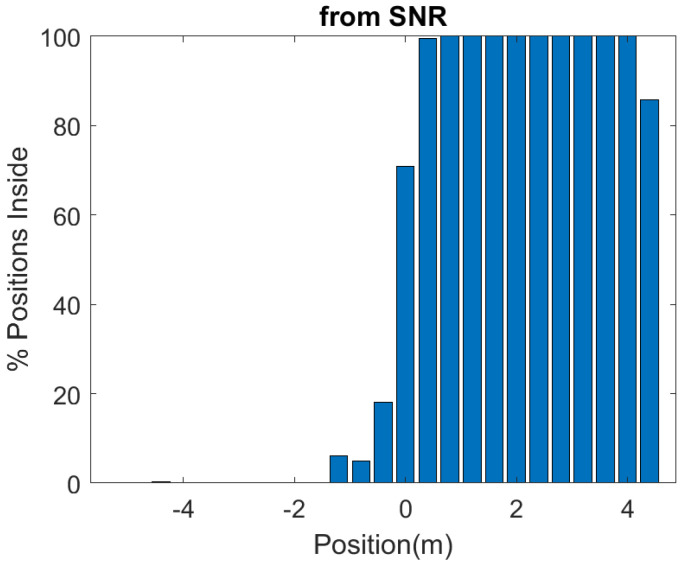
Percentage of cases in which the smart mask is classified inside, as a function of the position with respect to each of the receivers, obtained by comparing the measured SNR of each receiver at each position.

In order to investigate the performance of the positioning system, a number of measurements are done in a scenario in which both line-of-sight and multipath propagation effects must be taken into account. Figure 21 shows a schematic of the scenario. It consists of a corridor that allows access to the laboratory through the door. Each receiver is 2.4 m away from the door and 1.5 m above the ground. The transmitter is placed on top of the door. The entrance has two doors, one of which is made of metal and is kept closed. RSSI and SNR measurements are performed and recorded in each receiver in sets of backscattered packets measured every 40 cm along the corridor and the entrance of the laboratory, as shown in the diagram in Figure 21. A total of 1000 measurements are done at each position with the smart mask worn by one person. Figure 22 and Figure 23 show the average RSSI and SNR values measured at each receiver (receiver 1 outside and receiver 2 inside) and the error bars show the standard deviation obtained. From these data, the decision rule is applied for each measurement cell. Figure 24 and Figure 25 show the percentage of cases in which the RSSI or SNR measured at the receiver located in the laboratory is greater than that measured by the receiver located outside. Values close to 0% were found corresponding to the positions furthest from the receptor. The result agrees with the measured RSSI and SNR values that reach their minimum levels, close to the sensitivity of the receiver. These cases can be ignored if they are considered to be outside the cover range. Again, slightly better accuracy is observed when using SNR measurements instead of RSSI measurements. Near the door, the accuracy is ~30% due to the measurement uncertainty and the random nature of propagation. Therefore, it can be estimated that the resolution is about one range cell (±0.4 m), which is enough for this application. Therefore, the proposed decision rule to classify the relative position with respect to the door can be carried out without the need of a training procedure or the use of a specific propagation model.

To investigate the fluctuations of the RSSI (or SNR) depending on the position, a comprehensive empirical model has been developed. Propagation models applied to wireless signals within buildings have been extensively studied in different contexts such as cordless phones and wireless local area networks (WLANs) [42,43]. The proposed model considers the effect of multipath propagation due to reflections on walls, floor, and large objects, the shadowing or blockage of the transmitter and receiver due to the proximity of the body, and the attenuation due to walls and doors along the path. The model introduces an additional attenuation to the receiver power given by (10)

(14) $$L\left(dB\right)=L_{obs}\left(dB\right)+L_{multipath}\left(dB\right)+L_{blockage}\left(dB\right)$$

where $L_{obs}$ considers attenuation due to propagation through walls or other materials, and $L_{multipath}$ considers the attenuation due to the multipath interference, which is modeled using the two-slope model:

(15) $$L_{multipath}\left(dB\right)=10\cdot \left(n_{T}-2\right)\cdot log\left(1+d_{T}/R_{0T}\right)+10\cdot \left(n_{R}-2\right)\cdot log\left(1+d_{R}/R_{0R}\right)$$

where $n_{T}$ and $n_{R}$ are the path loss exponents for distances greater than the breakpoint distance [42], and $R_{0T}$ and $R_{0R}$ are the backscatter to the transmitter and the backscatter to the receiver paths, respectively. This model does not consider any obstacle in the first Fresnel region and the path loss exponent is close to 2 (or under 2 due to the corridor behaviour, equivalent to a wave-guide). On the other hand, a path loss exponent larger than 2 is expected when the distance is higher than the breakpoint. To account for the shadowing effect of the transmitter or receiver due to the proximity of the body, an increasing attenuation has been introduced as the backscatter approaches the transmitter or receiver:

(16) $$L_{blockage}\left(dB\right)=10\cdot n_{bT}\cdot log\left(1+R_{1T}/d_{T}\right)+10\cdot n_{bR}\cdot log\left(1+R_{1R}/d_{R}\right)$$

where $n_{bT}$ and $n_{bR}$ are exponent coefficients that control the level of attenuation, and $R_{1T}$ and $R_{1R}$ determine the range extension for the blockage of the transmitter and the receiver, respectively.

**Figure 26:**
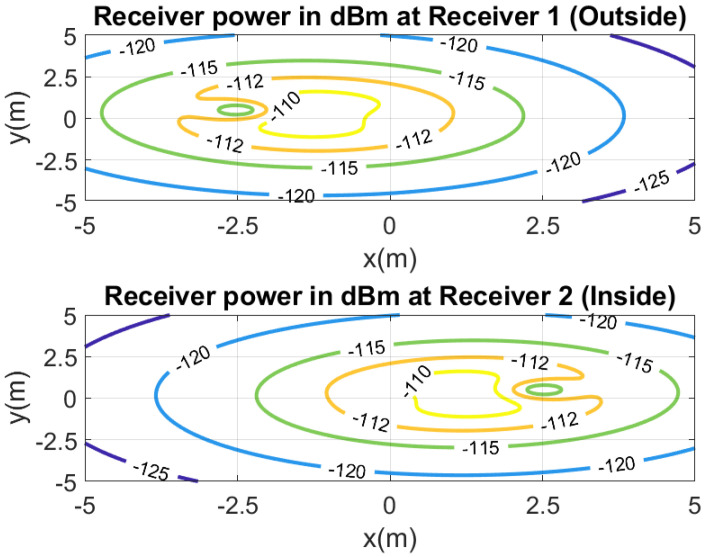
Simulated received power (RSSI) at the receiver located inside (**top**) and outside (**bottom**).

**Figure 27:**
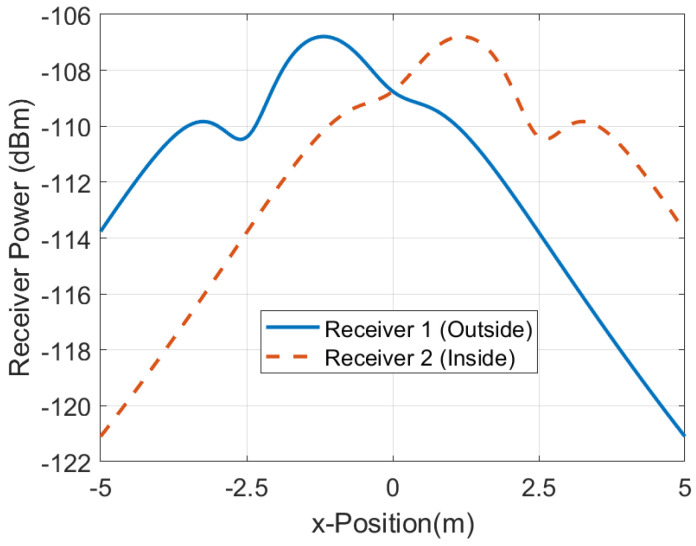
Simulated received power (RSSI) as a function of the x-position at y = 0.

Table 1 lists the empirical model parameters used in the simulations. Figure 26 shows a contour map of the received power (RSSI) simulated for each receiver for the same distances between transceivers and heights than in the scenario of Figure 21. Figure 27 shows a cut for y=0. Despite the limitations of the model, it justifies the evolution obtained in the measurements performed in this scenario. These figures show the ability of the model to predict the shadowing around the transmitter and receivers, and show the convenience of the decision rule (13) based on the highest RSSI (or SNR) that permits to predict if the person has crossed the gate or not(position x=0). Considering a deviation in the RSSI measurements of 2 dB, the resolution is in the order of ±0.5 m, which is in agreement with the experimental results.

## 4. Discussion

In this section, a comparison with other technologies (conventional and presented in the literature) is given. The comparison is summarized in Table 2. There are different commercially available methods for measuring body temperature. Body temperature measurement depends on the accuracy of the thermometer, the measurement site, and the skills of the person measuring the temperature. The aim is to measure core temperature, which is defined as the temperature of the large blood vessels in the internal organs and the brain. Temperature measurement using a pulmonary artery catheter is considered standard. However, this method is difficult to perform, and it is considered an invasive measurement method. Therefore, other methods and parts of the body such as the esophagus, rectum, armpits, and eardrum are used to measure body temperature [44]. Esophageal and rectal measurements are both generally considered valid [45] but are not suitable for non-contact or continuous readings. Therefore, methods for measuring body temperature from measurements performed on its surface are considered non-invasive and are preferable for continuous measurements or home use.

Body temperature is measured on the surface of the skin and results from the ambient temperature and the temperature inside the body. The main problem is that it depends on factors such as blood circulation, activity and external temperature, and serves as a regulator of the body’s temperature. While the core body temperature remains mainly constant, the surface temperature of the human body can vary considerably (approximately 28–37 °C). Depending on the ambient temperature, there can be big differences between the temperature of the interior and the surface of the body. The temperatures in the hands and extremities decrease several degrees in cold environments.

There are different ear or tympanic measurement devices commercially available. The most common are manual handguns, but recently there are also available portable over-the-ear devices for professional healthcare and for sports monitoring (e.g., from Cossinuss GmbH, Munich, Germany).

Axillary temperature measurement is a commonly used method. Modern sensors are based on a thermistor placed in the armpit, which is a place where the temperature remains stable and less influenced by the ambient temperature. Devices designed for continuous measurement are attached with the help of adhesive tape and can record the temperature and send the information via Bluetooth to a mobile application. Commercial examples of this technology are FeverSmart (model WT701, iMobile Healthcare LLC, Philadelphia, PA) and iThermonitor (model WT701, Railing Medical Company, Beijing, China). The main problem of these devices is given by the limitation of arm movements to prevent them from being disconnected or interfering with the measurement, as well as being problematic for measuring the temperature of babies.

In addition to those places where the body surface temperature is similar to the core temperature (such as armpits or eardrums), there are other strategies based on estimating the core temperature obtained from heat flux measurements, such as considered in this work.

The zero-heat-flux (ZHF) method is a non-invasive method [53] that is commercially available, for example, 3 M commercializes the model SpotOn (3 M, St. Paul, MN). Zero-heat-flux thermometers consist of a heater and two thermometers separated by a heat insulator. The heater is controlled to maintain the two temperatures identical. When these conditions are met, an isothermal tunnel from the core body to the skin surface is created. At this point, the subdermal temperature can be measured approximately 1 to 2 cm below the surface of the skin. In well-perfused parts of the body (such as the lateral forehead), the temperature of the tissue below the surface of the skin approaches the core body temperature. This non-invasive method is a useful tool for monitoring brain temperature. However, the current consumption associated with the heater makes this solution unsuitable for battery-powered or wearable devices. In addition, the patient must remain still to avoid the disconnection of the probes on the forehead.

As described in this work, dual heat flux sensors are used as an alternative to ZHF sensors with similar accuracy [31]. These sensors are installed also in the forehead and are commercially available for clinical use (e.g., Tcore product from Dräger, Lübeck, Germany). For instance, in [32,51], a dual heat flux sensor has been integrated into a headband that allows data to be transmitted to the mobile phone with Bluetooth.

Infrared thermometers are a cheaper solution than infrared camera-based thermal scanners and are widely used to measure forehead temperature to examine people. A comparison of the accuracy of infrared thermometers with a tympanic sensor has been presented in [54]; it shows that there are fixed offsets that depend on the site where the measurement is made (forehead or wrist), and therefore a suspicious person with fever requires a double check. In addition to the accuracy, the other issue arises from the need for a person to perform the measurement and, consequently, the system is not fully automatic.

In the last years, some research groups (e.g., Gaetano Marroco et al.) have developed epidermal temperature sensors based on UHF RFID tags [47,48,49,50]. The accuracy depends on the RFID integrated circuit, which embeds the temperature sensor and the RFID communication. These ICs have poorer sensitivity than conventional RFID ICs without sensing capability due to the extra energy required to bias the analog-to-digital converters as well as the sensors. In addition, losses introduced by the body limit the read range of these epidermal tags to a few meters. A prototype for the measurement of axillary temperature using passive RFID has been proposed in [47]. In [50], a dual sensor based on a dual UHF IC embedded in a UHF RFID tag has been proposed to improve the estimation of the body temperature. Although the cost of epidermal tags is low, the readers are expensive and justified if the entire blockchain over RIFD is deployed for other applications (e.g., to track the real-time location of assets, employees, or customers), limiting the application for small users.

Recently large investigation can be found about battery-less sensors based on energy harvesting Near-Field Communications devices [55]. These RFID tags obtain energy from the RF field to bias low-power microcontrollers and sensors. Contactless epidermal sensors based on battery-less Near-Field Communications (NFC) [55] integrated into textile has been recently investigated in [52]. This approach solves the requirement of a specific reader because a mobile with NFC can be used as a reader, but the read range is limited to few centimeters and gives the measurement of the shell temperature instead of the core temperature. Therefore, the accuracy and utility for screening depend on the site where the tag is attached; when the tag is in the armpit, measurements can be accurate, but this site is not very practical for automatic screening applications.

The system proposed in this work has the advantages of embedding the core temperature sensor in a face mask as well as being fully automatic. On the other hand, the estimation of the relative location can be measured using low-cost infrastructure based on commercial LoRa transceivers. Therefore, an expensive reader and the installation of additional wearable devices are not required. The communication based on backscattering extends battery lifetime. The main drawback is the low data rate due to the mechanism based on backscattering of LoRa packed that avoids the use of custom readers (e.g., SDR receivers). Therefore, for screening applications, basic information can be sent to avoid the amount of data (e.g., few bits to encode the temperature level, a bit to indicate the correct mask installation, a bit to indicate the usage time of the mask, and few bits to encode the breathing rate). The cost of the mask electronics is on the order of 5 € and a reusable future commercial device can be designed, changing the contaminated mask after use.

## 5. Conclusions

This work has proposed a system (addressed as smart mask) for long-range non-invasive wireless core body temperature and breath rate monitoring. It takes advantage of the mandatory use of the face mask in the interiors to integrate into it a temperature and breath rate monitoring system without requiring the use of another wearable (bracelet or headband). The implementation of a body temperature and breath control of people using face masks on a door is proposed in order to prevent entering persons with COVID-19-compatible symptoms. In addition, it can be detected if the person is located inside or outside the control point and its moving direction (if enters or exits) in order to implement a people counter and an occupant registration.

The system relies on backscatter communication as an alternative to other wireless communication system such as a Bluetooth. The latter sometimes requires to authenticate and also some infrastructure to estimate the location using proximity beacons (e.g., the location sensors and smartphones). The proposed backscattering system is implemented using low-cost LoRa devices. The high sensitivity of LoRa receivers allows for the detection and collection of information at enough range distance (a few meters). A proof-of-concept prototype has been designed, which detects the core temperature by means of a dual heat flux sensor and breath rate by means of measuring the exhaled temperature flow using an NTC. The core temperature sensor output has been compared to the measurement of the temperature in the armpit, obtaining a small difference in the accuracy, but it is enough for non-contact and autonomous screening applications. In addition, the multiple temperature measurements and the breathing rate estimation allow for both verifying correct face mask placement and usage time, avoiding the use of complex identification systems based on cameras and on computer vision. The smart mask also allows for the detection of coughing episodes from the sudden increase in thermistor temperature.

Other sensors could be integrated into smart masks, especially those used in industrial environments that are characterized by their larger size. Consequently, sensors such as those used to detect the presence of toxic gases (e.g., carbon monoxide or flammable gases) could be added to smart face masks. The transmission rate of the system is limited by the time between LoRa packets. Therefore, the amount of information to transfer is limited. To overcome this issue, in applications where the sensor can be applied to monitor the temperature or breathing during large periods, data can be saved in the memory of the microcontroller and downloaded later for processing. On the other hand, other wireless transmission systems such as Bluetooth Low Energy (BLE) can be employed to transfer the data.

## Figures and Tables

**Table 1:** Indoor propagation model parameters.

Parameter	Symbol	Value
Attenuation due to obstacles	$L_{obs}$	15 dB
Path loss exponent for transmitter to backscatter path	$n_{T}$	3
Path loss exponent for backscatter to receiver path	$n_{R}$	3
Breakpoint distance for backscatter path	$R_{0T}$	1.5 m
Breakpoint distance for backscatter to receiver path	$R_{0R}$	1.5 m
Blockage exponent for the transmitter	$n_{bT}$	4.5
Blockage exponent for the receiver	$n_{bT}$	4.5
Extension of blockage distance in the transmitter	$R_{1T}$	1.5 m
Extension of blockage distance in the receiver	$R_{1T}$	1.5 m

**Table 2:** Body temperature measurement methods.

Ref.	Technology	Advantages	Disavantages
Com.	Infrared Thermometer	Cheap	Operator needed. Close detection. Affected by external environment and conditions. Affected by body thermoregulation.
Com.	Thermo-scanner	Remote detection	Expensive. Affected by external environment and conditions. Affected by body thermoregulation.
Com.	Tympanic Thermometer	Cheap. Estimate of core temperature.	Operator needed. Close detection.
Com.	Axillary Thermometer	Cheap. Estimate of core temperature.	Close detection.
[46]	Zero-flux method	Estimate of core temperature.	Commercial available but wired. Power consumption for the heater.
[47,48,49,50]	UHF RFID	Cheap epidermal tag.	Expensive reader. Measurement depend on the position. Can be affected by external environment.
[32,51]	BLE and Dual Flow heat	Wireless measurement. Estimate of core temperature. Mobile as a reader.	Need a head band. Does not measure breathing or location. Requires authentication. Long-range.
[52]	NFC	Cheap. Mobile as a reader.	Measures the skin surface temperature. Measurement depend on the position. Affected by external environment and conditions. Affected by body thermoregulation. Short-range.
This work	LoRa Backscatter	Cheap. Estimation of core temperature. Estimation of breathing rate. Location. Uses commercial LoRa transceivers.Integrated in a mask. Long-range. Cough detection.	Battery-assisted. Low data transfer.

Com.: Commercial available.

## Data Availability

The data presented in this study are available on request from the corresponding author.
